# Can Assessing Physical Activity Liking Identify Opportunities to Promote Physical Activity Engagement and Healthy Dietary Behaviors?

**DOI:** 10.3390/nu13103366

**Published:** 2021-09-25

**Authors:** Patrice A. Hubert, Megan Mahoney, Tania B. Huedo-Medina, Tricia M. Leahey, Valerie B. Duffy

**Affiliations:** Department of Allied Health Sciences, University of CT, Storrs, CT 06269-1101, USA; Patrice.hubert@uconn.edu (P.A.H.); megan.m.mahoney@uconn.edu (M.M.); tania.huedo-medina@uconn.edu (T.B.H.-M.); tricia.leahey@uconn.edu (T.M.L.)

**Keywords:** physical activity, preference, college women, body size, dietary restraint, dietary behaviors, diet quality, physical exercise, diet, online survey

## Abstract

Improving our understanding of what physical activities are enjoyed and the factors that are associated with physical activity liking can promote participation in regular physical activity. We aimed to study physical activity (PA) liking in college women by modelling interactions between body size perception and dietary behaviors on PA liking, and by examining discrepancies between PA liking versus engagement on body size perception and dietary behaviors. Women (n = 251; 74% white) utilized an online survey to report their level of liking for PA types (scored into a PA liking index) and frequency of PA participation. They also reported their perceived body size, level of dietary restraint, and frequency of consuming foods (scored into a diet quality index). In multivariate analyses, a greater perceived body size was directly associated with lower PA liking and indirectly through greater dietary restraint but lower diet quality. Healthiest dietary behaviors were reported by women who both liked and engaged in PA. Women who reported high PA liking but low PA participation reported a higher dietary restraint and lower diet quality. These findings support the empowerment of women across all body sizes to identify physical activities that they enjoy. Health promotion efforts should encourage women to couple physical activity liking and engagement with a healthy level of dietary restraint and consumption of a healthy diet.

## 1. Introduction

Physical activity contributes to a healthy body weight as women age into early adulthood [1]. The Physical Activity Guidelines for Americans recommend that adults engage in at least 150 to 300 min a week of moderate intensity or 75 to 150 min a week of vigorous intensity aerobic physical activity and muscle-strengthening activities 2 or more days a week for health benefits [2]. Physical activity also supports academic achievement [3] and psychological well-being [4,5]. However, according to the Spring and Fall 2019 American College Health Association (ACHA) surveys, most college women (56–64%) do not meet the physical activity recommendations [6,7]. Pre-COVID-19, lack of physical activity coupled with living on a college campus with ready access to palatable but less healthy foods presented a challenge to maintaining a healthy body weight in college students [8]. COVID-19 restrictions, such as gym closures, mandated mask wearing, and social distancing, present a further challenge to physical activity and well-being, particularly in women [9,10]. Understanding ways to encourage physical activity in young women continues to be an essential task and remains an objective of ACHA’s Healthy Campus 2020 [11]. 

Attention to preferred physical activities encourages participation in physical activity [12,13,14,15], with the general belief that, overtime, liked physical activities will become ones that are sustainable. The present paper focuses on how body size perception and dietary behaviors influence physical activity liking. Previous research has supported the notion that physical activity participation is associated with body size perception but not in a consistent way [16,17,18]. Less is known about the relationship between body size perception and physical activity liking. Greater body size perception has motivated [19,20] and dissuaded [21,22] women from physical activity participation. Regular physical activity participation can promote positive body image perception [23,24]. This raises the question of whether greater liking of physical activity can support more frequent physical activity participation even if women perceive their body size as large. 

The relationship between body size perception and physical activity is also influenced by dietary behaviors, including cognitive control of eating (dietary restraint) and the healthiness of the diet (diet quality). While excessive dietary restraint has been of concern for disordered eating, appropriate levels of dietary restraint are associated with greater diet quality [25], successful body weight management, and health promotion [26,27]. College women, who may not be comfortable with public participation in physical activities, are more apt to control their dietary behaviors (dietary restraint/calorie restriction) when aiming to lose weight, without changing levels of physical activity [28]. However, in a longitudinal study, college women who presented weight concerns coupled with feelings of loss of control of eating and high hedonic value from food reported greater participation in physical activity over time [29]. This suggests that young women can use physical activity as a compensatory behavior for a poor or suboptimal dietary quality [30,31], or as a tradeoff when engaging in other unhealthy behaviors [32]. Additionally, physical activity has been seen to moderate dietary restraint and body weight changes. That is, appropriate levels of dietary restraint and adequate levels of physical activity support healthy weight maintenance [33]. Thus, assessing dietary behaviors can be key in understanding how to support sustainable physical activity behaviors. Participation in enjoyable physical activity should be encouraged among college women, with attention paid to the roles of body size perception and dietary behaviors on liking and frequency of physical activity.

In an online study conducted prior to the COVID-19 pandemic, we aimed to study physical activity liking among college women, including variability in physical activity liking related to body size and dietary behaviors as well as its relationship with frequency of engaging in physical activity. Survey assessment of liking can be a feasible way to identify motivators and barriers to exercise [34] and to promote preferred exercise patterns [35]. Research from our laboratory has shown that survey assessment of diet and physical activity likes and dislikes serves as a simple proxy of usual behaviors. Liking is part of a broader taxonomy used to describe complex behaviors, such as that described for dietary behaviors [36]. That is, reported liking of foods and beverages reflects usual food and beverage consumption as evidenced by associations with biomarkers of dietary intake [37], including in women [38] and young adults [39]. Therefore, we propose that using liking to measure physical activity can help to identify physical activity behaviors that are sustainable, as liking reinforces motivation, increasing adherence [40].

Our first aim was to model the liking of physical activity from body size perception and dietary behaviors (dietary restraint and diet quality). Few studies have specifically examined the interaction between body size perception and liking of physical activity [41], supporting a need for further examination. We hypothesize that greater body weight perception would be associated with lower liking of physical activity. Although there are mixed findings on the relationships between body size perception and frequency of physical activity [16,18,19,20,21,22], some studies report that greater body size perception may cause women to feel uncomfortable with physical activity [42,43,44], which could fuel lower liking of physical activity. Regarding dietary behaviors, we hypothesize that there are competing influences on the relationships between body weight perception and physical activity liking. That is, young women may be more willing to change dietary behaviors than physical activity in the presence of greater body size perception or concerns [26], whereas adolescent women without weight concerns report less healthy diet behaviors [45]. The greater focus on dieting may compel less interest and engagement in physical activity.

Our second aim extends the examination physical activity liking by comparing liking and reported frequency of engaging in physical activity. Previously, we demonstrated the greater ability to explain differences in dietary restraint and health outcomes in women who report that food and beverage liking and consumption are in agreement (e.g., high liking and high-frequency consumption) versus disagreement (e.g., high liking, low-frequency consumption) [38,46]. In regards to physical activity, we hypothesized that women who reported agreement between high liking and frequency of physical activities also would report the healthiest dietary behaviors and perceive the lowest or healthiest body size. Furthermore, we hypothesized that women who reported disagreement between liking and frequency of physical activities would report the least healthy behaviors. Findings from this study can help to identify how understanding liking of physical activity can be used to inform tailored interventions aimed at promoting physical activity in college women. 

## 2. Materials and Methods

### 2.1. Participants 

This was an observational, cross-sectional study using a convenience sample of 251 female students recruited through student newspaper postings to complete an online survey between February and March, 2018. The survey was open for all students; however, only 41 men participated. We found this number to be insufficient to reflect female vs. male differences in survey responses. There are significant gender effects on body size perception [47], dietary behaviors, and physical activity [48]. As a result, we only included responses from women in the analysis. The study was approved by the University Internal Review Board. The first page provided information about the study followed by yes/no consent to participate. There were no incentives or compensation provided to participants for survey completion.

### 2.2. Procedures

The online survey was programmed into Qualtrics (Provo, UT, USA) through the University and consisted of reported liking/disliking of physical activity, food and beverages, non-food-health-related behaviors; reported frequency of health behaviors (physical activity and diet); body size perception; dietary restraint; other health behaviors (perceived stress, and sleep); demographics; and additional student information. 

### 2.3. Liking of Physical/Sedentary Activities and Foods/Beverages

**Figure 1:**
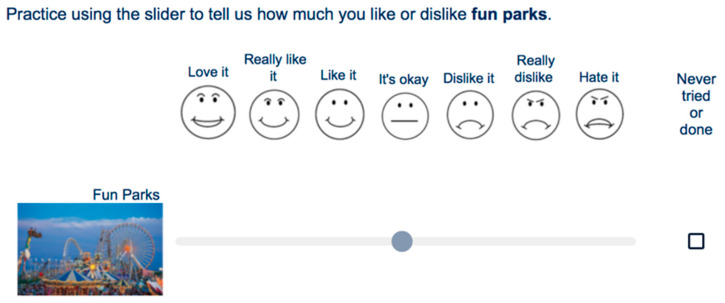
Sample question from the online survey with the hedonic facial scale, picture of the item, moveable scale, and ability to report “never tried or done”.

Participants were oriented to the scale by reporting level of liking of activities that are generally liked (winning the lottery, succeeding, fun parks), neutral (doing a routine chore), and disliked (running out of money, paper cut, waiting in traffic). These examples and the survey items were represented with both pictures and a circle indicator, which participants could move anywhere on the scale containing seven faces (Figure 1) labeled as “love it”, “really like it”, “like it”, “it’s okay”, “dislike it”, “really dislike”, and “hate it”. The Qualtrics program reported the distance measured from the scale center (0: “it’s okay”) to the ends of the bar (±100 “love/hate it)”, with intermediate values of “really dislike” (−75), “dislike it” (−50), “like it” (35), “really like it” (60), and “love it” (100). The participants could also mark “never tried/done” for any item. 

For the survey items, participants reported liking of 12 exercises under 4 domains of physical activity: aerobic (power walking, running at a slow/steady pace, running at a moderate pace, and sprinting), functional training (free weights, circuit training, and cable exercises), flexibility (yoga, stretching, and stability training), and resistance training (squats, deadlifts, bench press, and leg press). The physical activity items were content validated by experts in student health and physical activity assessment and pilot tested among university students [49]. The pilot study showed good internal reliability and variability among participants [49]. Three exercises were removed (participation in intramural sports, cable exercises, and deadlifts) due to low response rates. Participants also reported liking of 5 behavioral inclinations (working out alone/with a partner, taking the stairs, going to the gym, group fitness classes, and working up a sweat) and 4 sedentary activities (watching TV, taking the bus around campus, playing video games, and watching videos or movies on YouTube). These individual physical activity groups achieved or neared acceptable internal reliability (Cronbach’s α’s > 0.6).

Participants reported liking of several foods and beverages (protein powder, sports drinks, pre-packaged coffee drinks, energy drinks, milk, fruits, and vegetables) [49]. Items were chosen to capture food groups and sweetened beverages consumed by college students as seen in previous pilot testing [49] and our findings with young adults [39].

The Physical Activity Liking Index (PAI) is comprised of the liking ratings for individual physical activities. These ratings are averaged into groups and theoretically weighted (group average × weight), and the weighted groups are then averaged into the PAI. The multiplier weights follow the American College of Sports Medicine guidelines: aerobic exercise (+3), functional training (+1.5), flexibility training (+2), resistance exercises (+2.5), sedentary activities (−3), and behavioral inclinations (+3). Higher weights represent increased cardiometabolic health benefits. Through content validation, aerobic exercise was deemed to be the most influential factor on health and provided the highest weighting [49]. This physical activity liking index score (PAI) was tested for reliability and validity following the framework used to evaluate the Healthy Eating Index [50,51]. This framework outlines criteria to test the validity and reliability of survey-generated indexes for health behaviors. For validity, the index should give maximum and minimum scores to behaviors, score variation among individuals, and distinctions between groups with known differences in behavior. For reliability, internal consistency is assessed by examining relationships among the index components and identifying which components have the most influence on the total score. This PAI score met the requirements for validity (outlined further in the Results Section) and neared sufficient internal reliability (Cronbach’s α = 0.65).

### 2.4. Frequency of Physical/Sedentary Activities and Foods/Beverages

For physical activity engagement, the participants responded to questions about their level, frequency, and intensity of physical activity. For physical activity level, responses were either sedentary, lightly active, moderately active, or extremely active. For frequency and intensity, the participants identified the number of days per week that they worked out, the number of exercise repetitions in a set, and the length of workout (minutes). These frequency and intensity responses were multiplied together to create a physical activity exposure score, with good reliability (Cronbach’s α = 0.801). 

For food and beverage intake as well as measures of diet quality, the participants reported the frequency of consuming fruits, vegetables, fast food, sweets or salty snacks, and sweetened beverages. Responses were either never, couple times/month, weekly, daily, or more than one time/day. Each response was recoded into a value from 0 to 5 and then theoretically weighted based on the 2015 Dietary Guidelines [52] by multiplying the corresponding category: eating fruits (+5); eating vegetables (+7); eating fast food (−3); eating sweets or salty snacks (−5); and drinking sugary, sweetened beverages (−7). This method of weighting has been validated in similar studies using liking surveys to create diet quality index scores among college students, with higher weights reflecting a healthier diet and reduced cardiometabolic risk factors [39,53]. The resulting values were then added together to create a dietary index score (Cronbach’s α = 0.54). Scores of >4 aligned with healthier diet quality and score ≤−7 aligned with less healthy diet quality. Participants also reported their diet quality as poor, fair, good, very good, or excellent for comparisons with the calculated diet quality index [54]. 

### 2.5. Body Size Perception

**Figure 2:**
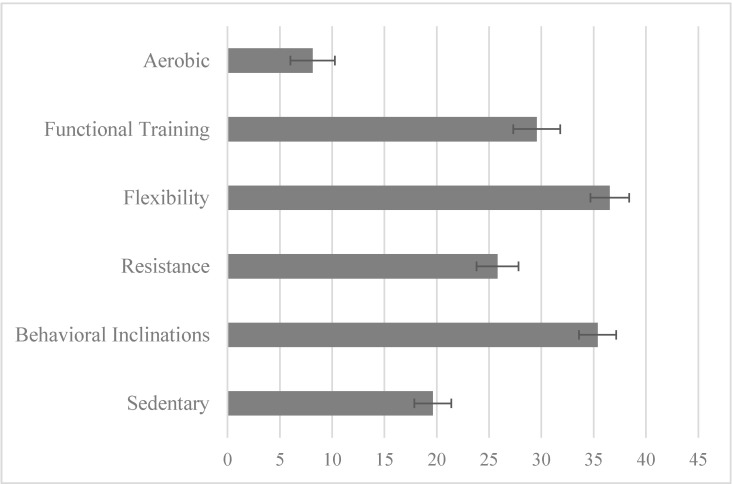
Average liking of groups of physical activities in the physical activity liking index, where participants rated the activities on a bidirectional hedonic scale (0 = “it’s okay” to ± 100 “love/hate it”) and intermediate values of “really dislike” (−75), “dislike it” (−50), “like it” (35), “really like it” (60), and “love it” (100).

**Figure 3:**
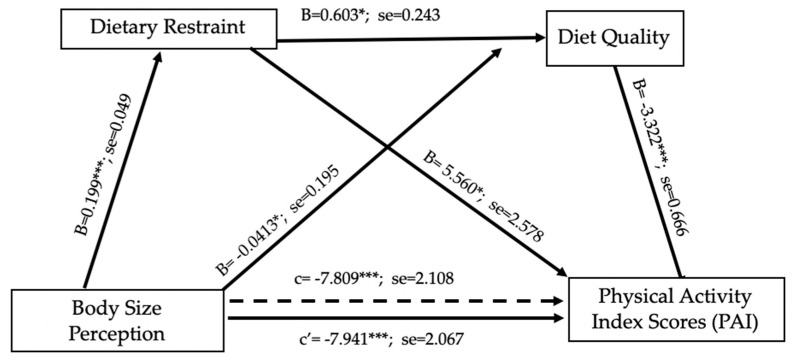
Serial multiple path analysis of dietary restraint and dietary quality in the relationship between perceived body size and physical activity index scores with non-standardized beta values. * *p* < 0.05, *** *p* < 0.001. Greater body size perception associated with decrease in liking of physical activity (PAI), greater dietary restraint, and lower diet quality. Higher dietary restraint associated with higher diet quality and higher liking of physical activity. Higher dietary quality associated with lower liking of physical activity. Simultaneously dietary restraint and diet quality indirectly influenced liking of physical activity in relation to body size perception.

**Figure 4:**
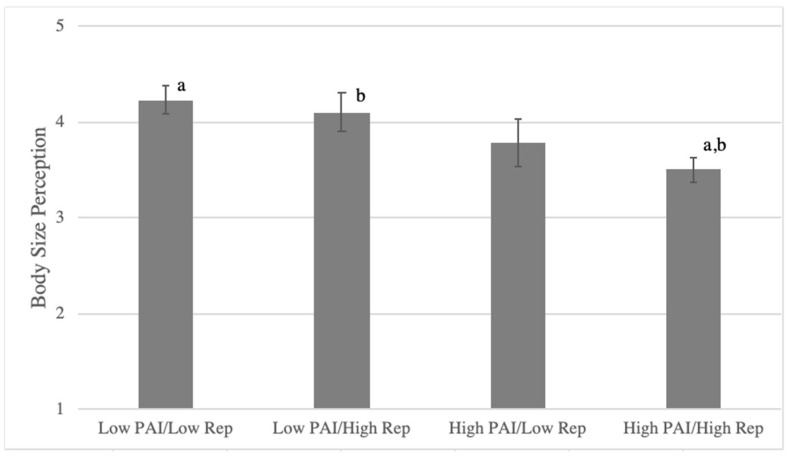
Body size perception among concordant/discordant groups in PAI (physical activity liking index) versus reported frequency of physical activity in college women; according to the Figure Rating Scale [55] 1–2 = underweight, 3–4 = normal weight, 5–6 = overweight, and 7–9 = obese. Matching letters denote significant difference (*p* < 0.05).

**Figure 5:**
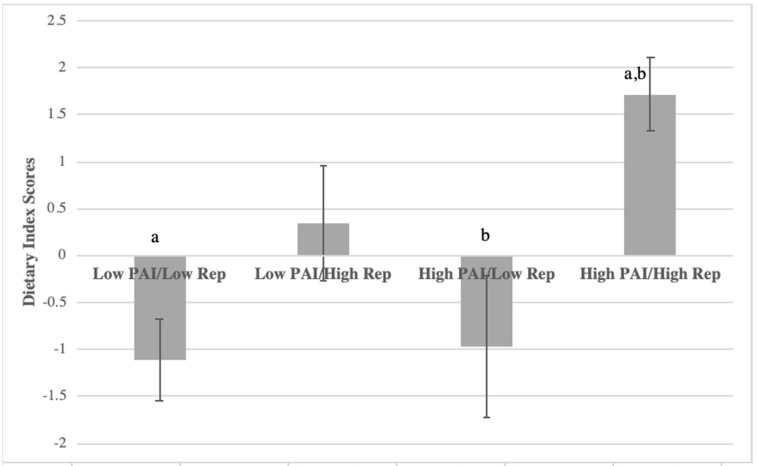
Average dietary index scores among concordant/discordant groups in PAI (physical activity liking index) vs. reported frequency of physical activity in college women. Dietary index scores (diet quality) were the sum of weighted consumption fruits, vegetables, sweets/salty snacks, and sugary beverages where >4 = healthier scores and <−7 = less healthy scores. Matching letters denote significant difference (*p* < 0.05).

**Figure 6:**
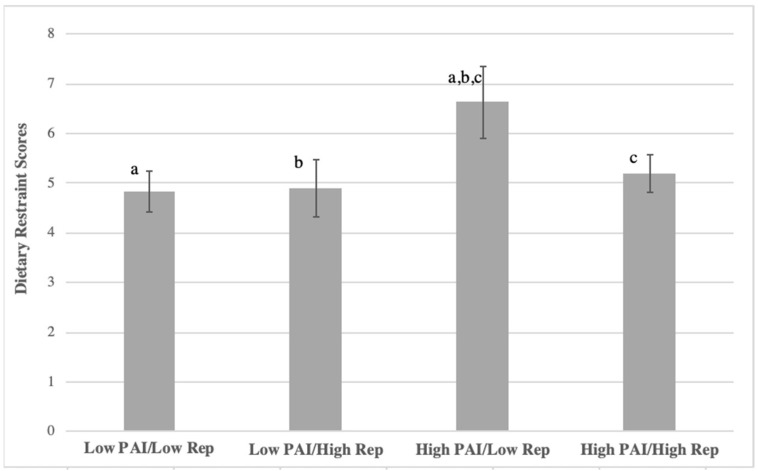
Average dietary restraint scores among concordant/discordant groups in PAI (physical activity liking index) vs. reported frequency of physical in college women. Scores were the sum of responses to three restraint questions, ranging from 0 = never to 12 = daily on each. Matching letters denote significant difference (*p* < 0.05).

Participants were questioned about their body size perception in two ways: via use of the Figure Rating Scale [55] and a single question regarding perception of being underweight, normal weight, overweight, or obese. The Figure Rating Scale asks participants to select the figure that best represents their current body size from 9 male or female body figures, which increase in size from underweight to obese (1–2 = underweight, 3–4 = normal weight, 5–6 = overweight, and 7–9 = obese). This scale has been validated as an easy tool used to measure body size perception and body dissatisfaction in college women [56,57,58], with good test–retest reliability [56]. For the present study and following the figures in the Figure Rating Scale [55], women were categorized as underweight/normal weight (body size Figures 1–4) and overweight/obese (body size Figures 5–9). 

### 2.6. Dietary Restraint

Dietary restraint was assessed using three items from the Dutch Restrained Eating Scale [59]. These items were used in pre/post-survey assessment of The Body Project, a dissonance and healthy weight eating disorder program aimed at improving body image in young women [60] delivered at our university. The 3 chosen questions primarily regarded diet behaviors influenced by weight with high item-test correlation coefficients among past students who participated in the program. Different from the original Likert response format, items were responded to on a five-point frequency scale (0–4) ranging from never to daily, and participants responded to the following questions: (1) (Eat Less) If you put on weight, did you eat less than you normally would? (2) (Weight Decide Eating) Did you take into account your weight in deciding what to eat? (3) (Avoid Eating) How often did you try not to eat between meals because you were watching your weight? Restraint scores could range from 0 to 12. Responses were summed to obtain the dietary restraint score with good reliability (Cronbach’s α = 0.788). 

### 2.7. Statistical Analysis

Data were analyzed using SPSS statistical software for Mac (version 24, Chicago, IL, USA) with Process v3.4 (2019); significance was set at *p* < 0.05. Power analysis variables undergoing parametric testing (e.g., PAI) were assessed for normality. Descriptive analyses were performed with and without outliers to determine effect. Results with outliers removed (PAI scores greater than −100; *n* = 5) are presented, as these responses also had abnormal values for the pleasant/unpleasant items used for scale orientation. Testing of the reliability and validity of PAI was adapted from the methods used to evaluate the Healthy Eating Index [50,51]. Paired sample t-tests examined the differences in liking of physical activity groups among participants. Reliability was tested with Cronbach’s alpha (α) and correlational statistics. Concurrent criterion validity of the PAI was tested by comparing values to self-reported physical activity (category and exposure score), diet quality score, perceived body size (categorical and Figure Rating Scale), and dietary restraint score. 

Linear regression was used to conduct a path analysis of three variables (body size perception, dietary restraint, and dietary index scores) on physical activity liking (PAI). Based on the regression-based approach proposed by Hayes [61], analysis of variance (ANOVA) was used to test for differences in the concordant and discordant groups in reported physical activity and liking. Variables tested included perceived body size, dietary quality, and dietary restraint. PAI scores and reported physical activity levels were split at the median to form concordant (low liking/low reported and high liking/high reported) and discordant (low liking/high reported and high liking/low reported) groups. These groups allowed for the identification of individuals who were health promoting (high liking/high reported), health seeking, or trying to change behaviors (low liking/low reported), and individuals who may need behavior change intervention (high liking/low reported and low liking/low reported). The assumptions of ANOVA were tested, including evaluating normality and outliers of dependent variables at each level of the independent variable. Levene’s test was used for equality of variances at each level of the independent variable. 

## 3. Results

### 3.1. Descriptive Findings

The completion time for the survey ranged from 5 to 10 min. Table 1 displays the characteristics of the study sample. Most of the women were between 19–22 years of age, identified as white, and reported light-to-moderate physical activity. 

Of the total sample who completed the survey, 23.4% reported they were in a health-related major, while others reported a variety of majors in science, engineering, business, and liberal arts fields. Just over 25% reported as being overweight/obese based on the two measurements of body size perception, which showed a moderate correlation (rho = 0.61 (*p* < 0.001). There was good variability in the questions concerning dietary restraint, from infrequent behaviors to weekly and daily behaviors. Dietary index scores ranged from −13 to 8.4, with an average score of 0.26. The correlation between the categorical rating of dietary quality and dietary index scores was significant (r = 0.582, *p* < 0.0001), with women who reported poor diet quality averaging between −4 and −5 for the dietary index and women who reported very good/excellent diet quality averaging between 3 and 4. Thus, most of the women reported healthy diet quality but with room for improvement, as only 19% reported the consumption of multiple servings of fruits and vegetables per day.

### 3.2. Physical Activity Liking and the Physical Activity Liking Index (PAI)

Overall, physical activity was generally liked by the study sample (Figure 2). The average liking of physical activity groups (aerobic, functional, flexibility, resistance, and behavioral inclinations) was higher than average liking of sedentary behaviors (mean(s): 28.1 ± 1.4 SE vs. 19.6 ± 1.8 SE; t(225) = 3.86; *p* < 0.001). Reported liking of individual physical activity groups averaged between a neutral rating (“it’s okay”) and liking rating, demonstrating a positive preference for physical activity in this sample, ranging from the lowest average liking of aerobic activities (nearing “it’s okay”) to flexibility exercises and behavioral inclination categories averaging at “like it.” Liking of aerobic exercises was significantly different from that of all other exercise groups (functional training t(232) = −7.70, *p* < 0.001; flexibility: t(250) = 10.88, *p* < 0.001; resistance: t(238) = −6.11, *p* < 0.001; general Exercise t(250) = −14.47, *p* < 0.001). 

Each physical activity liking group showed significant correlations (*p* < 0.001) with PAI. Sedentary and flexibility exercises (rho = −0.335 and 0.376, respectively) had the least influence on overall PAI score, while general exercise behaviors had the most influence (rho = 0.818). The PAI showed significant variation and a normal distribution (W(251) = 0.91), with scores ranging from −84 to 168, (mean = 41.23 ± 2.91 SE). Exploratory factor analysis indicated two dimensions within the PAI score (active and less active) that accounted for >60% of variability.

The PAI scores were higher in those who self-reported to be moderately and extremely active as a categorical rating (F(3, 251) = 38.29, *p* < 0.0001). There also was discordance between those who reported low or high liking and frequency of activity categories, suggesting other motivations or intent for engaging in physical activity. The PAI was also positively correlated with reported physical activity behavior as a continuous composite variable (physical activity exposure score) (r = 0.611, *p* < 0.001). 

The PAI scores were lower in participants who perceived themselves as heavier, significant for perceived categorical ratings (normal/underweight vs. overweight obese; F(1251) = 3.81) but not reaching significance for those perceiving themselves as overweight/obese on the Figure Rating Scale (F(1251) = 2.59, *p* = 0.11). Similarly, for associations with diet, PAI scores were higher in participants who reported higher diet qualities, significant for both categorical response (F(4, 251) = 9.38, *p* < 0.0001) and for the continuous dietary index score (r = 0.339, *p* < 0.0001). However, unlike body size perception and diet quality, dietary restraint was not significantly correlated with PAI scores (r = 0.086, *p* = 0.173).

### 3.3. Multivariate Modeling of Physical Activity Liking

The simultaneous effect of body size perception, dietary restraint, diet quality (measured by dietary index scores), and PAI scores was modeled based on individual associations (Figure 3). The total effect (c = −7.809, SE = 2.11, t = −3.70, *p* < 0.001) of body size perception on PAI scores was significant, indicating greater body size perception associated with lower PAI scores. Body size perception had a positive direct effect on dietary restraint (b = 0.199, SE = 0.049, t = 4.025, *p* < 0.001) and a negative direct effect on diet quality (b = −0.413, SE = 0.195, t = −2.115, *p* < 0.05), implying that greater body size perception is associated with greater dietary restraint and a lower diet quality. The direct effect of dietary restraint as the first mediating variable on the second mediating variable of diet quality (b = 0.603, SE = 0.243, t = 2.484, *p* < 0.05) was also significant, suggesting that diet quality increased alongside dietary restraint. A review of the direct effects of mediating variables on PAI scores showed that the effects of dietary restraint (b = 5.56, SE = 2.578, t = 2.157, *p* < 0.05) and diet quality (b = 3.322, SE = 0.666, t = 4.99, *p* < 0.001) were significant. A greater level of dietary restraint was associated with higher PAI scores, but the reverse relationship was seen with diet quality, where low diet quality indicated higher PAI scores. This could suggest compensatory behaviors in this study sample. 

When body size perception and the two mediating variables were entered simultaneously into the model, the direct effect of body size perception on physical activity liking index scores was found to be significant (c’ = −7.941, SE = 2.067, t = −3.842, *p* < 0.001) but slightly lessened, demonstrating evidence of serial mediation. This suggested that the combined effect of dietary restraint and diet quality indirectly influenced liking of physical activity related to body size. Some of the associations between greater body size perception and lower liking of physical activity were explained by a greater level of dietary restraint but lower diet quality.

### 3.4. Relationships between Physical Activity, Body Perception, and Dietary Behaviors

While liking and frequency of physical activity showed a significant correlation, there were women who were concordant (33.1% low in both; 39.8% high in both) and discordant (16.3% low liking/high frequency; 10.8% high liking/low frequency) in these measures of physical activity behaviors. Differences in body size perception, diet quality (dietary index scores), and dietary restraint were tested with ANOVA among participants who were concordant and discordant in liking (PAI) versus frequency of physical activity. Body size perceptions were significantly different among the concordant/discordant groups (F(3251) = 5.12, *p* < 0.005) (Figure 4). The lowest average body size was perceived by those who had both higher liking and frequency of physical activity, significantly lower than those who reported either both low liking and frequency of physical activity or low liking and high frequency of physical activity (all *p* < 0.05). 

Similarly, the highest diet quality (dietary index scores) was observed among participants who reported high physical activity liking and frequency of physical activity (F(3, 251) = 8.901, *p* < 0.001; Figure 5), and the dietary index scores of such participants were significantly greater than those in the two groups with low frequency of physical activity (*p* < 0.005). 

Overall, dietary restraint was not significantly different among the concordant/discordant groups (F(3, 251) = 1.628, *p* = 0.184) (Figure 6). However, the greatest dietary restraint was seen in those with high physical activity liking and low frequency of physical activity. Post hoc analysis showed that this group significantly differed from the group with both low liking and frequency of physical activity. 

## 4. Discussion

This paper describes an innovative approach to studying physical activity and diet behaviors through asking what is liked. We aimed to describe how liking of physical activity is associated with body size perception and dietary behaviors as well as interactions with reported frequency of physical activity. The convenience sample of collegiate women reported a low level of liking of physical activity, being lightly to moderately physically active, having a moderate level of dietary restraint, fair diet quality, and nearly 30% perceiving an overweight/obese body size. Serial mediation modeling revealed that women who perceived greater body size reported tradeoffs between physical activity liking and dietary behaviors—heathier diets were associated with lower liking of physical activity, whereas reasonable levels of dietary restraint were associated with greater liking of physical activity. By examining the agreement and disagreement between liking and frequency of physical activities, women who reported high liking and high frequency of physical activities had the lowest perceived body size and the healthiest diet quality. The highest level of dietary restraint was seen in women with high liking but low frequency of physical activities. These findings support the need to promote enjoyable physical activities at all body sizes, to encourage enjoyable physical activities coupled with healthy dietary behaviors, and to identify barriers faced by women who like but do not participate in physical activity. 

Our physical activity liking measure was practical and novel. Recall of liking is cognitively simpler than recall of behaviors [62], which allowed a relatively quick assessment of multiple physical activities as well as foods and beverages. The novel physical activity liking index (PAI) acknowledges that a variety of physical activities support physical health, which is in accordance with the Physical Activity Guidelines for Americans, 2nd edition [2]. To our knowledge, there is no single physical activity measure that encompasses liking for a variety of physical activities. The PAI had acceptable psychometric properties as demonstrated by good variability and normal distribution in this sample of college women, acceptable internal reliability, and more than one theme in construct validity testing. The PAI was correlated with frequency of physical activity, which agrees with previous research [34,35] and supports the benefit of measuring liking of physical activity to address reasons for physical inactivity [34]. Comparing liking with the reported frequency of physical activities identified those with the healthiest dietary behaviors and potentially with the most sustainable physical activity behaviors. 

The intersecting relationships between liking of physical activity (PAI), body size perception, and dietary behaviors in the present study are consistent with those observed in previous reports [23]. We found that women with greater body size perception had lower liking of physical activity partially explained by higher, but reasonable levels of dietary restraint, as well as lower diet quality. Appropriate levels of dietary restraint may prevent unwanted weight gain and support healthy diet quality on college campuses that provide students with unlimited access to less healthy food throughout the day and night [8,26,27]. However, we observed opposite relationships between dietary behaviors and liking of physical activity. Dietary restraint positively predicted greater physical activity liking, suggesting the women were consistent with their health behaviors, while diet quality predicted lesser liking of physical activity, suggesting a disconnect in health behaviors. The highest level of dietary restraint and lowest diet quality were found among women who reported that they liked but did not engage in physical activity, identifying the need to balance the healthiness of diet and physical activity behaviors. College women tend to have compensatory health behaviors [30,31], as observed in the present study, where one behavior is used to replace another, especially when physical activity is not easily accessible [28]. 

Our findings are also consistent with the negative health impacts of perceiving an elevated weight [42,43]. That is, one being a higher weight or overweight does not always motivate behavior change [21,22]. It was suggested in a recent review that greater body perception causes psychological distress resulting from internal and societal weight stigmatization, which negatively impacts health promoting behaviors [42]. Simply perceiving oneself to have a greater body size can fuel feelings of shame and discourage physical activity engagement in young adults [43]. Decreased comfortability in public places [28] and internalized weight stigma [44] further hinder physical activity. Some women in the present study may have relied more on dietary restraint than physical activity to manage their greater perceived body size. However, dietary restraint efforts do not always translate to improved diet quality [26], which is consistent with the negative association between body size perception and diet quality in our sample. Compensatory behaviors, especially when influenced by body size misperception [17], do not sustain healthy behaviors [29] and may lead to unwanted weight gain [42]. Promoting education about healthy body size perception is important for the cultivation of healthy behaviors in young women [63]. Frequent physical activity participation promotes increased body satisfaction [64], a healthier body image [23,24], and reduced risk of disordered eating [23]. In our sample, women who both liked and participated in physical activity reported the lowest perceived body size and healthiest diets. 

There are some limitations to this study that are worth noting. Statistical limitations include the removal of outliers (<2%) and violation of the ANOVA assumption for normality. The sample was homogenous in race/ethnicity, limiting the generalizability of the findings. Body size perception may vary across different racial and ethnic groups [65], as may cultural beliefs about physical activity and dietary behaviors in women [66]. The survey was based on self-reported data. Although we suggest that liking is a reasonable proxy of behavior, bias and inaccuracy always exist in self-reported data. Social desirability may also be a limitation. Even through a discrete online platform, participants may have answered what they thought researchers wanted and in relation to the social norms of their peers reported on social media. The specific physical activities and exercises asked about on the liking survey may not capture the range of types that collegiate women engage in, which could have falsely lowered the PAI. However, the behavioral inclinations sub-score of the PAI were not specific to an activity type and showed significant correlation with the PAI. Only three questions from the Dutch Eating Behavior questionnaire were used, limiting the comparability to other studies. Lastly, our diet quality index score was based on a limited number of items and had a Cronbach’s α of 0.54, which is below the acceptable range and lower than the Healthy Eating Index, which is based on multiple items (Cronbach’s α = 0.68) [51]. However, diet quality is known to be multidimensional, and reliability may not be a necessary characteristic [51]. Our diet quality index score demonstrated a strong correlation with the reported diet quality from participants, suggesting further confidence in this index. 

Despite the limitations, our findings can be applied to support physical activity and healthy dietary behaviors among collegiate women. While our sample of women was a convenience sample, the participants represented multiple college majors and displayed sufficient variability in physical activity liking, dietary behaviors, and perceived body size to test the study aims and hypotheses. The perceived level of overweight/obese and reported dietary intake as well as the level of physical activity were consistent with U.S. pre-pandemic surveys of college women [6,7]. Measuring the liking and frequency of physical activity can promote sustainable physical activity engagement [34]. Emerging evidence from our laboratory suggests that motivating and reinforcing health promotion messages can be delivered online to college students based on their reported liking of diet and physical activities; these messages are reported to be relevant and useful [67]. This evidence expands the tailored message program that we previously conducted with children and parents [68,69]. These tailored messages can highlight specific areas of change prior to an intervention or counseling session and promote motivation and self-efficacy to change health behaviors [67,70,71]. Focusing tailored counseling and health messages on body misconceptions, healthy dietary behaviors, and enjoyable physical activity supports the overarching goal of health promotion.

## 5. Conclusions

This study utilized liking to feasibly measure physical activity in college women with a novel, valid, and reliable index that captured a variety of activity types. Multivariate modeling showed that women who perceived greater body size reported less liking of physical activity as well as less healthy dietary behaviors. Women who both liked and engaged in physical activities had a lower body size perception and healthier diet quality. The COVID-19 pandemic saw increases in both physical activity and sedentary behaviors among college students [10]. Assessing physical activity liking could help to improve our understanding of drivers and barriers of physical activity for tailored health counseling and interventions.

## Figures and Tables

**Table 1:** Characteristics of college women (*n* = 251).

**Age Group**	
17–18 years	18.3%
19–20 years	42.6%
21–22 years	31.5%
23+ years	7.6%
**Race**	
White	74.1%
Black	4.0%
Hispanic	8.4%
Other	13.5%
**Reported Physical Activity Level**	
Sedentary	8.0%
Lightly Active	35.9%
Moderately Active	46.2%
Extremely Active	10.0%
**Body Size Perception**	
*Categorical*	
Underweight	3.6%
Normal Weight	72.1%
Overweight/Obese	24.3%
*Figure Scale Rating* †	
Normal	71.3%
Overweight/Obese	28.7%
**Diet Quality (Self-Reported ††)**	
Poor	5.6%
Fair	28.3%
Good	36.3%
Very Good	27.1%
Excellent	2.8%
**Dietary Restraint questions †††**	
*Eat Less*	
Never	44.6%
Couple times/month	24.0%
Weekly	7.4%
Couple times/week	13.2%
Daily	10.7%
*Weight Decide Eating*	
Never	18.4%
Couple times/month	21.6%
Weekly	8.8%
Couple times/week	22.0%
Daily	29.2%
*Avoid Eating*	
Never	35.2%
Couple times/month	15.2%
Weekly	6.4%
Couple times/week	20.4%
Daily	22.8%

† Based on [55]; †† based on [54]; ††† three items from the Dutch Restrained Eating Scale: (1) Eat Less—“If you put on weight, did you eat less than you normally would?” (2) Weight Decide Eating—“Did you take into account your weight in deciding what to eat?” (3) Avoid Eating—“How often did you try not to eat between meals because you were watching your weight?” [59].

## Data Availability

The data presented in this study are available on request from the corresponding author.
